# Cystic lesions and their mimics involving the intrahepatic bile ducts and peribiliary space: diagnosis, complications, and management

**DOI:** 10.1007/s00261-024-04742-6

**Published:** 2024-12-26

**Authors:** Rachita Khot, Dhakshinamoorthy Ganeshan, Karthik M. Sundaram, Jena N. Depetris, Daniel R. Ludwig

**Affiliations:** 1 Radiology and Medical Imaging, University of Virginia, Charlottesville, VA USA; 2 Department of Abdominal Imaging, The University of Texas MD Anderson Cancer Center, Houston, TX USA; 3 Department of Radiology, Hospital of the University of Pennsylvania, Philadelphia, PA USA; 4 Department of Radiology, UCLA Health, Los Angeles, CA USA; 5 Mallinckrodt Institute of Radiology, Washington University School of Medicine, Saint Louis, MO USA

**Keywords:** MRI–MRCP, Peribiliary cysts, Caroli disease, Cirrhosis, Adult polycystic kidney disease, IPN-B

## Abstract

Biliary and peribiliary cystic lesions represent a diverse group of abnormalities, often discovered incidentally during imaging for unrelated conditions. These lesions, typically asymptomatic, necessitate precise imaging modalities to characterize their nature and determine subsequent clinical actions, such as follow-up imaging, biopsy, or surgical referral. The anatomic location of these cystic lesions, whether biliary or peribiliary, influences both diagnostic and prognostic outcomes. Biliary cystic lesions, such as mucinous cystic neoplasms, intraductal papillary neoplasms of the bile duct, and Caroli disease, require careful monitoring due to their propensity to develop malignancy. In contrast, peribiliary cysts are often associated with chronic liver disease and may indicate disease progression through a gradual increase in cyst size. Accurate differentiation of these lesions from other clinical entities that have overlapping features on imaging, such as microabscesses, bilomas, Langerhans cell histiocytosis, neurofibromatosis, and vascular anomalies such as cavernous transformation of the portal vein, is essential given the divergent management for each. This article focuses on intrahepatic biliary and peribiliary cystic lesions and their mimics, highlighting their imaging characteristics with an emphasis on magnetic resonance imaging and magnetic resonance cholangiopancreatography, differential diagnosis, potential associated complications, and clinical management.

## Introduction

Intrahepatic biliary and peribiliary cystic lesions are often asymptomatic, and clinically significant because of their potential to indicate underlying liver or biliary pathologies. These lesions, which can be either congenital or acquired, encompass a wide spectrum of conditions, from benign cysts to complex pathologies with malignant potential (Table 1). Understanding their nature, etiology, and clinical implications is important in effective patient management. Imaging is pivotal in evaluating these lesions, particularly given their nonspecific clinical presentation.

**Table 1:** Summary of intrahepatic biliary and peribiliary cystic lesions and their mimics, including their imaging appearance, complications, and management

Condition	US appearance	CT appearance	MRI appearance	Complications	Management
Peribiliary cysts	Multiple small anechoic cysts around the bile ducts	Hypodense cystic lesions without enhancement around the bile ducts	T1WI hypo- and T2WI hyperintense cysts around the bile ducts; nonenhancing on postcontrast images	Obstructive jaundice or cholangitis (rare)	Conservative management; treatment of complications
Von Meyenberg complex (VMC)	Small VMCs are hyperechoic; larger VMCs are usually hypoechoic or anechoic. May have comet-tail artifacts	Multiple, small nonenhancing, hypodense lesions	T2WI: hyperintense, scattered, tiny cysts T1WI: isointense or mildly hyperintense No biliary communication; “starry sky” appearance on T2WI	None	No specific treatment
Caroli disease	Multiple intrahepatic cystic or saccular dilatations of intrahepatic bile ducts Central dot sign on color Doppler	Hypodense cystic dilatation of intrahepatic bile ducts with the “central dot sign”	T2WI: cystic “string of pearls”; “central dot sign”; no enhancement on post-contrast T1WI	Cholangitis, biliary stone formation, biliary cirrhosis, increased risk for cholangiocarcinoma	Treatment of cholangitis and endoscopic intervention for stone formation
MCN-L	Large, thick-walled, anechoic, multilocular cyst; may have septations	Well-circumscribed, hypodense, multilocular cyst; thick wall, septation, rarely calcifications	T2WI: hyperintense cyst with thin septations T1WI: variable because of mucin content; septal enhancement	Malignant transformation to mucinous adenocarcinoma	Surgical resection due to malignant potential
IPN-B	Bile duct dilatation with or without mural nodule	Bile duct cystic dilatation with enhancing nodules, aneurysmal dilatation of bile ducts	T2WI: hyperintense cystic lesions communicating with bile duct on MRCP with or without enhancing mural nodules or septations; biliary duct dilatation	Risk of malignant transformation to cholangiocarcinoma	Surgical resection due to malignant potential
Intrahepatic biloma	Anechoic or hypoechoic fluid collections adjacent to bile ducts	Well-circumscribed, nonenhancing, hypodense collection	T2WI: hyperintense fluid collection adjacent to biliary tree T1WI: hypointense; nonenhancing	Infection, rupture, peritonitis	Conservative management, percutaneous drainage
Abscess	Complex cystic lesion with internal echoes, debris, and irregular walls	Low-attenuation lesion with peripheral rim enhancement (“rim sign”)	T2WI: hyperintense T1WI: hypointense with peripheral enhancement DWI: restricted diffusion	Sepsis, rupture, peritonitis, systemic infection	Antibiotics, drainage, surgery if complicated
Lymphatic malformations	Large, multiseptated, anechoic-hypoechoic lesions	Low-attenuation cystic lesion with thin septations	T2WI: hyperintense, multiseptated T1WI: hypointense with minimal to no enhancement	Mass effect; may mimic cystic tumors	Surgical excision or observation depending on size
Langerhans cell histiocytosis	Variable; may show focal hypoechoic or cystic lesions in the liver	Hypodense liver lesions, often heterogeneous; may enhance	Variable: T2WI: hyperintense lesions T1WI: isointense; may show enhancement on postcontrast	May cause biliary obstruction, hepatic fibrosis	Immunotherapy, chemotherapy, steroids, liver transplant
Neurofibromatosis	Usually appears as hypoechoic lesions	Hypodense; may show slight enhancement	T2WI: hyperintense masses, “target sign” appearance T1WI: iso- to hypointense; shows enhancement on postcontrast	May mimic biliary or peribiliaty cystic disease, risk of transformation to malignant peripheral nerve sheath tumor	Surveillance; surgical resection for symptomatic lesions
Vascular entities	Cavernous transformation: complex, serpiginous, anechoic or hypoechoic lesions with flow on color Doppler Aneurysms and pseudoaneurysm: round, anechoic with color flow	Cavernous transformation: serpiginous hypodense cystic lesions; enhancement postcontrast Aneurysm: hyperdense with enhancement	Cavernous transformation: T2WI: hyperintense; enhancement postcontrast Aneurysm: T2WI: signal void in center T1WI: isointense, enhancing postcontrast	Portal hypertension, bleeding, rupture	Surgical intervention, stenting, or embolization depending on type

*DWI* diffusion-weighted imaging, *IPN-B* intraductal papillary neoplasm of the bile duct, *MCN-L* mucinous cystic neoplasm of the liver, *MRI* magnetic resonance imaging, *T1WI* T1-weighted imaging, *T2WI* T2-weighted imaging

Intrahepatic biliary and peribiliary cystic lesions often coexist with other hepatic abnormalities, further complicating their clinical evaluation. Imaging not only aids in distinguishing these lesions from other hepatic conditions but also guides therapeutic decision-making, ensuring that patients receive the most appropriate care based on the specific characteristics of the lesions.

This review focuses on intrahepatic biliary and peribiliary cystic lesions, their imaging characteristics, differential diagnosis including a discussion of their mimics, potential associated complications, and management. It emphasizes the role of magnetic resonance imaging (MRI) and magnetic resonance cholangiopancreatography (MRCP) in enhancing diagnostic accuracy and guiding clinical management.

## Anatomy of the bile ducts and peribiliary region

The biliary system is primarily categorized into intrahepatic and extrahepatic bile ducts, distinguished by their anatomic location in relation to the liver hilum. The smallest intrahepatic bile duct branches start from the canals of Hering. Emerging from the canaliculi are ductules that lead to interlobular bile ducts, which combine to form septal ducts, collectively known as small intrahepatic bile ducts (IHBDs). The large IHBDs consist of area, segmental, and right and left hepatic ducts. At the hepatic hilum, the left and right hepatic ducts merge to form the extrahepatic common hepatic duct. The common hepatic duct joins with the cystic duct from the gallbladder to become the common bile duct, which ultimately combines with the pancreatic duct and drains into the duodenum through the papilla of Vater [1, 2].

**Fig. 1:**
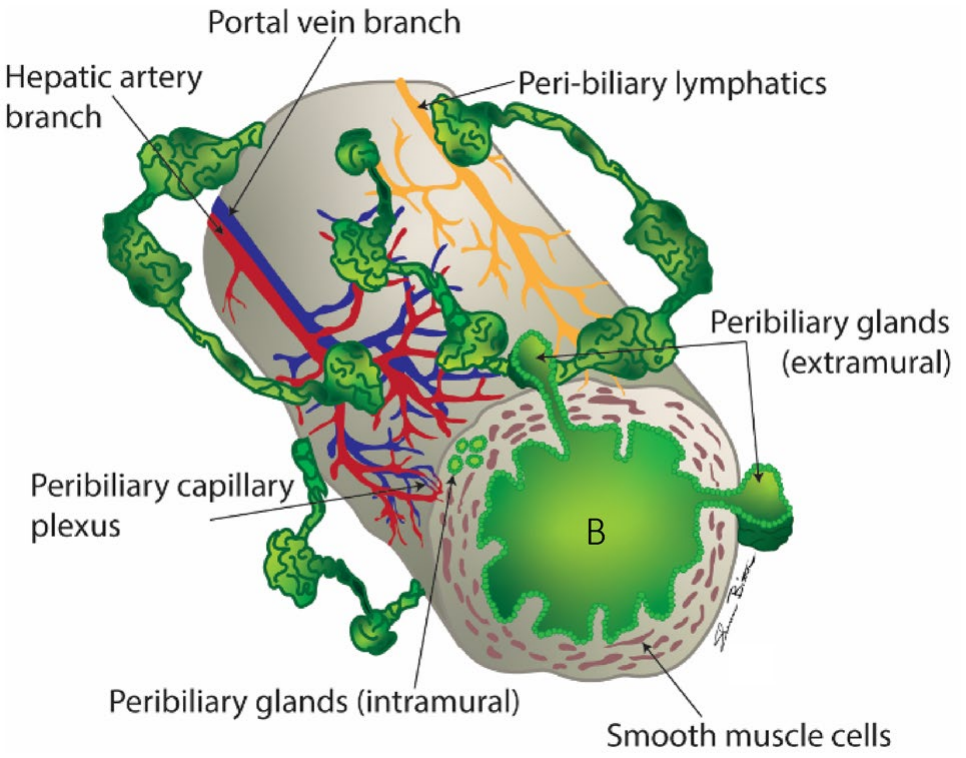
Illustration shows the bile ducts (B) and anatomic structures in the peribiliary space. The peribiliary space is an anatomical area surrounding the biliary ducts, consists of loose connective tissue containing nerve bundles, lymphatics, blood vessels, isolated smooth muscle cells, and the extramural peribiliary glands (PBGs). The PBGs are either intramural or extramural, depending on their location within the bile duct wall. It is the extramural PBGs that can dilate to form peribiliary cysts

The peribiliary space, an anatomic area surrounding the biliary ducts, consists of loose connective tissue containing nerve bundles, lymphatics, blood vessels, isolated longitudinal and circular bundles of smooth muscle cells, and the extramural peribiliary glands (PBGs, Fig. 1) [3]. The PBGs are either intramural or extramural, depending on their location within the bile duct wall. It is the extramural PBGs that can dilate to form peribiliary cysts. These glands are connected to the bile duct lumen via small canals to excrete their secretions. The extramural PBG canal also forms an interconnecting network with adjacent PBGs, providing a bypass in cases of biliary obstruction or stenosis. They are surrounded by the peribiliary vascular plexus, supplied by small branches of the hepatic and gastroduodenal arteries and drained by hepatic sinusoids and small veins into the portal vein [3]. The functions of PBGs include producing and secreting mucus to protect the biliary epithelium, digestive enzymes, and substances for local immunity; absorbing bile; relieving intraluminal pressure during obstruction; and serving as a site for biliary epithelial regeneration and as a reservoir for multipotent stem and progenitor cells [3, 4].

## Imaging modalities

Imaging is essential in the diagnosis, management, and surveillance of biliary and peribiliary cystic lesions. Given the relatively lower cost, wide availability, and lack of radiation, ultrasound (US) is commonly used to evaluate for biliary dilation. Although this may be helpful in the initial assessment, most patients require further imaging with computed tomography (CT) or MRI. CT is the workhorse in diagnosing and guiding management of many peribiliary cystic lesions, given its high spatial resolution, greater accessibility, and ability to acquire images rapidly. Besides providing greater detail and higher diagnostic accuracy, CT is well suited to depict complications and the presence of any coexisting pathologies.

MRI with MRCP is an excellent imaging modality for detailed evaluation and characterization of biliary and peribiliary cystic lesions. It is often considered superior to other modalities in specific clinical scenarios requiring high soft tissue resolution and delineation of the biliary system, including identifying communication with bile ducts. The absence of ionizing radiation makes MRI a safer option for repeated imaging. Despite some limitations, including longer acquisition times, higher costs, and relatively limited access, MRI with MRCP provides exquisite details on peribiliary cystic pathologies and often serves as a roadmap in patients warranting surgical intervention. Extracellular gadolinium-based contrast agents are commonly used, but hepatobiliary agents such as gadoxetate disodium can be used to demonstrate the continuity of a peribiliary lesion with the bile duct [5, 6].

## Biliary and peribiliary cystic lesions

### Peribiliary cysts

The exact etiopathogenesis of peribiliary cysts is not yet clear but is thought to be related to obstruction and cystic dilatation of extramural PBG [7]. These are frequently associated with chronic liver disease, including cirrhosis, portal hypertension, autosomal dominant polycystic liver disease, portal vein thrombosis, and liver transplantation [8–10]. Peribiliary cysts are classically located in the hepatic hilum and adjacent to the proximal intrahepatic bile ducts. About 90% of patients present with multiple cysts, but up to 10% have a solitary cyst. The cysts are periportal in distribution, following the bile duct and vary in size (1–55 mm) [9].

**Fig. 2:**
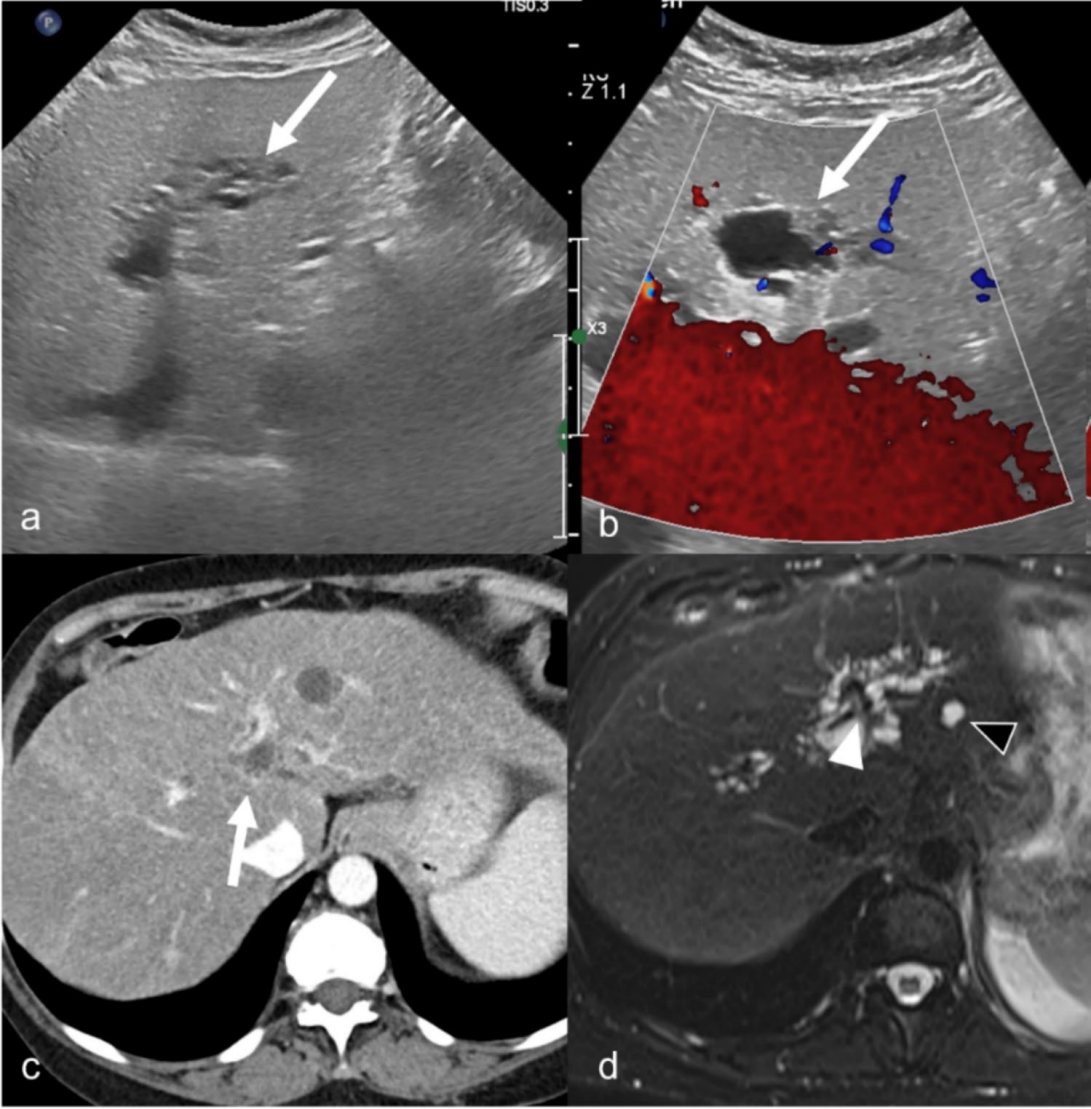
Peribiliary cyst in a 54-year-old female patient with a history of alcoholic cirrhosis and worsening of disease over time. Grayscale (**a**) and color Doppler (**b**) ultrasound images show cystic changes without flow on color Doppler (arrows). Axial contrast-enhanced CT shows cystic changes along the portal vein (arrow). MRI Axial T2-weighted fat-saturated (**d**) image shows multiple small T2-weighted hyperintense cysts along the portal vein (white arrowhead) and a simple cyst (black arrowhead). The MRI better depicts the extent of peribiliary cyst than the other imaging modalities

Imaging is useful in the diagnosis and management of peribiliary cysts [9–12]. On US, peribiliary cysts typically appear as small, well-defined, anechoic structures with posterior acoustic enhancement. Identification of thin septa between the cysts can help to distinguish these from dilated bile ducts [9, 10]. On CT, peribiliary cysts are seen as multiple, small, homogenous, low-attenuation foci distributed adjacent to the bile ducts and hepatic hilum. On MRI, these cysts are T1-weighted hypointense and T2-weighted hyperintense, without post contrast enhancement. MRCP can be helpful and demonstrate a lack of communication with bile ducts (Fig. 2). Delayed imaging with gadoxetate disodium can be used to confirm a lack of ductal connection.

**Fig. 3:**
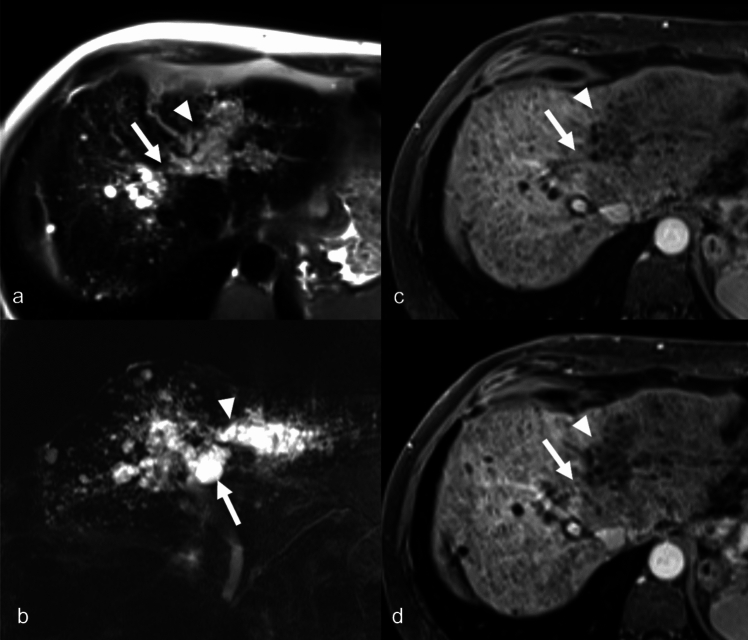
Peribiliary cysts mimicking cholangiocarcinoma in a 54-year-old female with history of hepatitis C, alcoholic cirrhosis, and chronically elevated total bilirubin (4.3 mg/dL) presenting with concern for a suspicious left medial lobe liver lesion on a pre-transplant CT chest with contrast. Axial T2-weighted image (**a**) demonstrates a cirrhotic liver that appears dark due to iron deposition. Cystic lesions are visualized along the right and left (arrowhead) ducts extending from the hilum (arrow). The cystic conglomerate in the left lobe was the area of concern on prior CT. On thick-slab MRCP (**b**), the lesions appear more round rather than as dilated ducts consistent with peribiliary cysts. Late arterial phase (**c**) and delayed phase (**d**) MRI shows the cystic region in the left hepatic lobe (arrowhead) and the hilar region (arrow) with no abnormal enhancement to suggest underlying mass. CA-19 was normal and brushings by ERCP were negative

Although these are often asymptomatic and diagnosed incidentally on imaging, some patients, especially those with larger and progressive cysts, can rarely develop complications such as obstructive jaundice or cholangitis. Peribiliary cysts can sometimes be mistaken for biliary dilation secondary to cholangiocarcinoma. However, they can be readily distinguished from cholangiocarcinoma using thin-slice T2-weighted and dynamic contrast-enhanced MRI, as there is no underlying mass associated with peribiliary cysts (Fig. 3). Surgical or endoscopic interventions may be necessary if there is coexistent biliary obstruction, though the majority of cases require no intervention or specific follow-up.

### Von Meyenberg complexes

Von Meyenberg complexes (VMCs), also known as multiple bile duct hamartomas, arise from ductal plate malformations of smaller interlobular bile ducts [13, 14]. On histopathology, these are seen as clusters of small, irregularly shaped bile ducts lined by cuboidal epithelium, occasionally with surrounding dense collagenous stroma. Given their embryological origin, VMCs can be associated with other ductal plate malformation diseases, such as polycystic kidney disease (PKD), Caroli disease, Caroli syndrome, and congenital hepatic fibrosis. Autopsy studies have reported VMC prevalence of approximately 6% in adults and 1% in children [15].

**Fig. 4:**
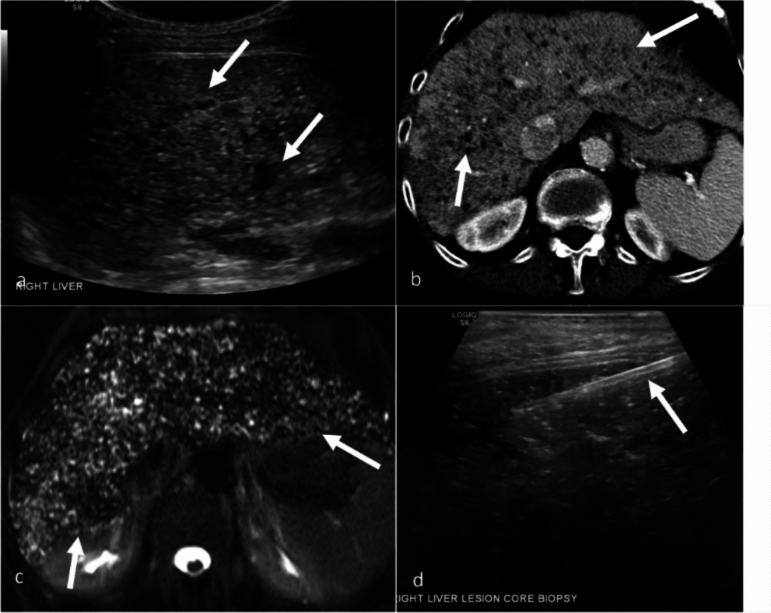
Von Meyenberg complex in a 91-year-old-female patient with a colonic mass and liver lesions on CT. Grayscale US image (**a**) shows innumerable cysts and hyperechoic lesions in the liver (arrows). Axial contrast-enhanced CT (**b**) shows tiny cysts scattered throughout the liver and no enhancement. Axial MRI T2-weighted fat-saturated (**c**) shows innumerable T2-weighted hyperintense cysts. The imaging findings on MRI are diagnostic, and biopsy is rarely indicated. US-guided biopsy (**d**) in this case confirmed the findings

VMCs are almost always discovered incidentally when imaging is performed for unrelated indications. VMC typically presents as numerous small lesions ranging 1 to 30 mm in size and are often seen in peripheral, subcapsular, or periportal locations, although solitary lesions may occur. On US, VMC has variable imaging features depending on their size [14, 16]. Small VMCs tend to be hyperechoic because the back walls of these tiny cysts are echogenic reflectors and may cause diagnostic dilemmas, mimicking metastases [13, 17]. Larger VMCs are usually hypoechoic or anechoic. Comet-tail or reverberation artifacts may be seen. On CT, VMCs are seen as multiple small subcentimeter hypoattenuating lesions in the liver [18]. Typically, no enhancement is present, although a thin peripheral rim enhancement may be seen related to the enhancement of the adjacent liver parenchyma [10]. However, given the multiplicity and tiny size of the lesions, the possibility of hepatic metastases can be difficult to exclude, especially in patients with a known history of primary malignancy [10, 13, 14, 17]. MRI helps differentiate VMCS from other differential diagnoses, characteristically demonstrating high signal on T2-weighted (T2W) sequences with longer echo time and no associated restricted diffusion [19]. In some patients, thin peripheral rim enhancement or mural nodules may be seen [19, 20]. A biopsy may be considered for definitive diagnosis in patients with equivocal imaging findings, especially those with a known or suspected extrahepatic malignancy (Fig. 4). VMC does not require any follow-up or treatment; however, an associated ductal plate malformation such as autosomal dominant PKD-associated congenital hepatic fibrosis may require treatment for the underlying liver disease [19, 21].

### Caroli disease

Caroli disease is a rare congenital fibropolycystic disorder, sometimes referred to as a type V choledochal cyst. It is characterized by segmental, saccular, or fusiform dilatation of predominantly large intrahepatic bile ducts, which may occur in isolation (Caroli disease) or more commonly in association with PKD and congenital hepatic fibrosis (Caroli syndrome) [22]. Although hepatic involvement is typically diffuse, segmental involvement has also been reported. The resulting biliary dysgenesis and dilatation predispose to bile stasis, which can lead to biliary stone formation, recurrent cholangitis, or biliary cirrhosis [23]. The classic clinical presentation of Caroli disease includes biliary colic, episodes of recurrent cholangitis, and manifestations of portal hypertension in later stages of the disease.

**Fig. 5:**
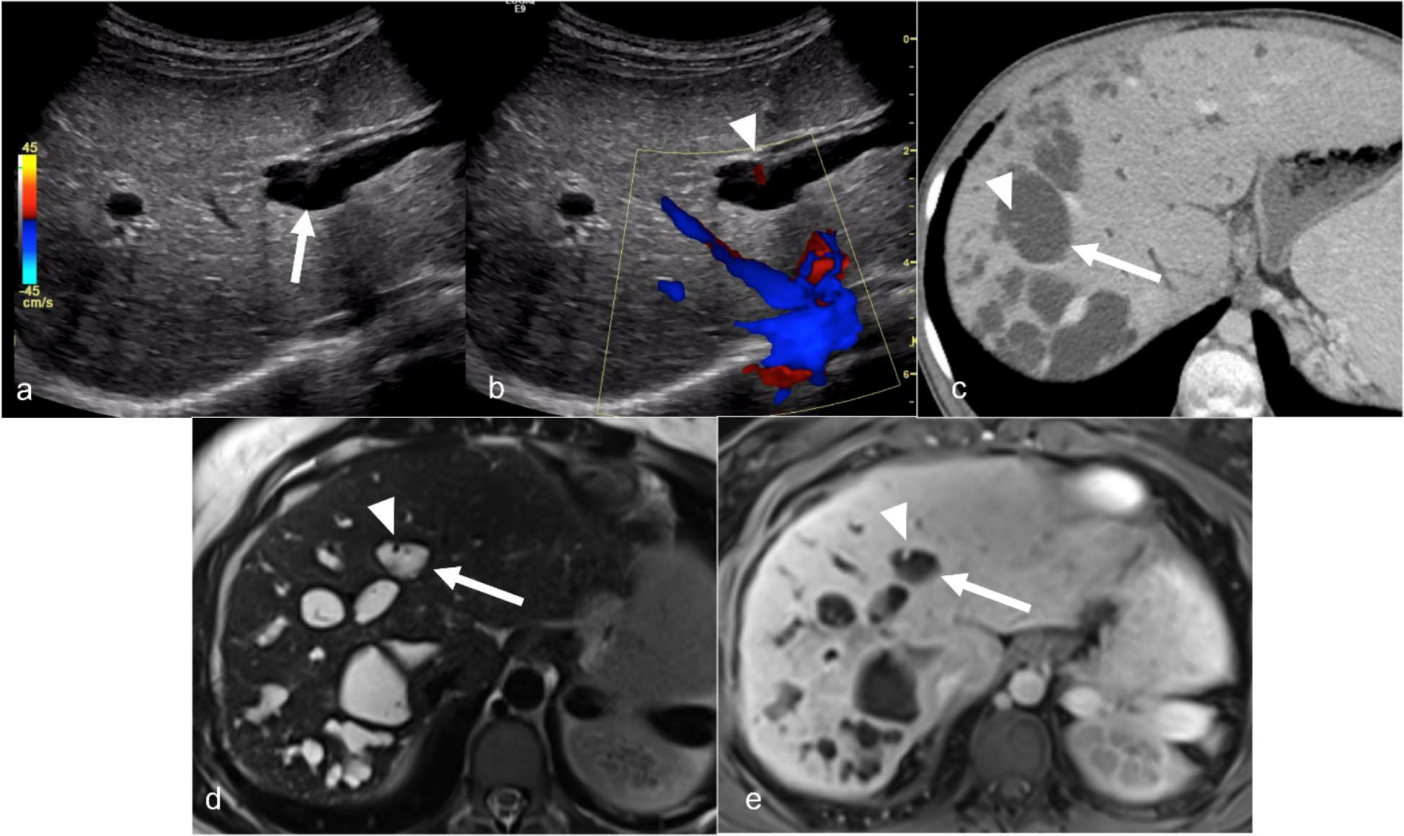
Caroli disease in a 39-year-old patient with abdominal pain. Grayscale (**a**) and color Doppler (**b**) ultrasound images show a dilated bile duct (arrow) with a dot of blood flow (arrowhead). Axial contrast-enhanced CT (**c**) shows multiple cysts (arrow) with a central dot sign of enhancing portal vein branch (arrowhead). Axial MRI T2-weighted fat-saturated (**d**) and postcontrast T1 (**e**) images show multiple T2-weighted hyperintense cysts (arrow) with a small focal area of hypointensity (arrowhead), which shows enhancement, corresponding to a portal vein branch on postcontrast image (arrowhead, **e**)

On US, cystic dilatation of the intrahepatic bile ducts and the “central dot sign,” representing portal venous and hepatic arterial branches surrounded by dilated bile ducts, are seen [23]. Biliary stones or sludge can sometimes be identified when present. On CT or MRI with MRCP, the findings are similar and can be used for additional characterization of the biliary tree with increased sensitivity and specificity for the diagnosis (Fig. 5). MRCP is useful for clarifying the relationship of the areas of cystic dilatations to the rest of the biliary tree with the use of heavily T2W MRCP sequences or hepatobiliary contrast agent excretion into the bile ducts and identifying associated intrahepatic biliary stones. Additionally, cross-sectional imaging modalities allow for the detection of associated PKD and other manifestations of cirrhosis, including findings of portal hypertension (splenomegaly, ascites, or portosystemic shunting) [9].

Complications of Caroli disease relate to ongoing biliary stasis, including biliary stone formation, recurrent episodes of cholangitis, and biliary cirrhosis [24]. Treatment for Caroli’s disease includes symptomatic treatment of cholangitis with antibiotic therapy, medications for cholestatic liver disease with ursodeoxycholic acid, and endoscopic intervention for complications of biliary stasis and stone formation [24]. Because of episodes of recurrent cholangitis and bile duct inflammation, these patients are reportedly at a 100-fold increased risk for the development of cholangiocarcinoma compared with healthy individuals; therefore, routine surveillance is warranted, and partial liver resection versus orthotopic liver transplantation may be required for definitive oncosurgical treatment [22].

## Mucinous cystic neoplasm of the liver

Mucinous cystic neoplasm of the liver (MCN-L), previously known as biliary cystadenoma or cystadenocarcinoma, is a rare mucin-producing bile duct tumor that is most commonly benign but classified by the World Health Organization fifth edition of Digestive System Tumors as a premalignant lesion [25–27]. This cystic neoplasm occurs almost exclusively in middle-aged female in the fourth to fifth decades of life and is more commonly located in the left hemiliver. Histologically, it is characterized as a cyst-forming epithelium neoplasm lined by cuboidal, columnar, or flattened mucin-producing epithelium overlying ovarian-like stroma, a key distinguishing pathologic feature and similar to its counterpart, the mucinous cystic neoplasm of the pancreas [26].

Small sized MCN-L are typically asymptomatic therefore, they are more often diagnosed at a large size with symptoms related to the mass effect on the adjacent structures, including abdominal bloating, pain, early satiety, or a palpable mass in the right upper quadrant.

**Fig. 6:**
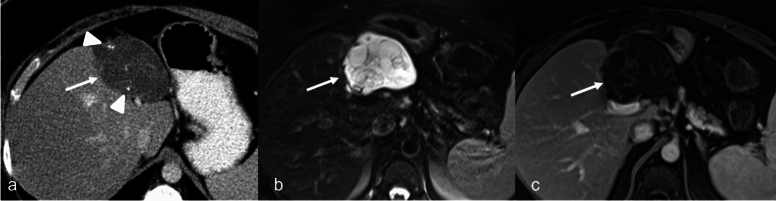
Mucinous cystic neoplasm of the liver in a 59-year-old female patient with a history of endometrial cancer and a liver lesion noted on staging CT. Axial CT (**a**) shows a lobulated cystic lesion with septations and small areas of calcifications (arrowheads). Axial MRI T2-weighted fat-saturated (**b**) and postcontrast T1 (**c**) images demonstrate a cystic lesion with multiple septations, seen as linear hypointensity within in the cyst (arrow). The septations demonstrates smooth enhancement post contrast and no evidence of nodularity

MCN-L presents as a large unilocular or, more commonly, multiloculated, multiseptated liver lesion. On US it is seen as an anechoic cystic lesion. Mural calcifications or nodularity may be present; these are considered features associated with underlying dysplasia or malignancy. CT and MRI are helpful when the lesions are large and difficult to visualize completely with US. On CT, MCN-L is seen as a large, well-defined, multiloculated, low-density cystic lesion. Septa arising from the cyst wall without associated wall indentation is a sensitive feature for distinguishing these from complex cysts [28]. Mural calcification is a more specific imaging feature of MCN-L [26]. On MRI, they are T2 hyperintense with variable T1-weighted image (T1WI) because of the mucinous contents (Fig. 6). When calcification is present, it is seen as a low signal intensity along the wall or septa. Contrast-enhanced CT and MRI may show enhancement of internal septations, mural nodules, or solid components when present. MRCP is also useful for delineating the relationship of the lesion to the biliary tree and the presence of associated upstream biliary ductal dilatation [26, 29].

Although more than 90% of MCN-L lesions are benign, they are considered precursor lesions; therefore, low-grade dysplasia, high-grade dysplasia, or rarely associated invasive carcinoma may occur. Because of the risk of malignant transformation, complete surgical resection or enucleation is typically recommended, with an excellent prognosis after complete surgical resection. This is favored over other therapeutic interventions, such as percutaneous aspiration, surgical fenestration, and marsupialization, which have higher recurrence rates. Surveillance for suspicious features is also often reasonable, especially if the differential includes a complex cyst or the patient is a borderline surgical candidate [26, 30].

## Intraductal papillary neoplasm of the bile duct

Intraductal papillary neoplasm of the bile duct (IPN-B) is a premalignant lesion of the bile ducts characterized by papillary proliferation of neoplastic biliary-type epithelium. It shares imaging and histologic features with the intraductal papillary mucinous neoplasm (IPMN) of the pancreas [31]. However, IPN-B differs because only 30% to 40% exhibit mucin hypersecretion; therefore, the term *IPN-B* is used instead of *IPMN-B* [26]. This condition is more prevalent in East Asian populations, likely because of higher incidences of *Clonorchis sinensis* infection and hepatolithiasis, with chronic biliary inflammation playing a crucial role in its pathogenesis [32, 33]. Many patients with IPN-B are asymptomatic, though they may present with pain, jaundice, or cholangitis [34].

IPN-B exhibits a range of histologic subtypes, including the pancreaticobiliary, oncocytic, gastric, and intestinal, each contributing to its diverse histologic behaviors [26]. The lesion’s appearance on imaging largely depends on the amount of mucin hypersecretion relative to solid papillary growth. Extensive mucin production can lead to aneurysmal dilation of the bile duct with or without visible papillary tumor in the duct [35]. This dilation can be segmental or diffuse, often showing stringlike filling defects corresponding to concentrated mucin bundles, frequently evident on T2W MRI or MRCP images, known as the “thread sign” [36]. Whereas CT and US can also display duct dilation and papillary tumors, MRI with MRCP is significantly more sensitive, especially when combined with hepatobiliary phase imaging, which improves mucin detection. Mucin is suspected when there is filling defect in the bile duct on hepatobiliary phase imaging [26, 37, 38].

**Fig. 7:**
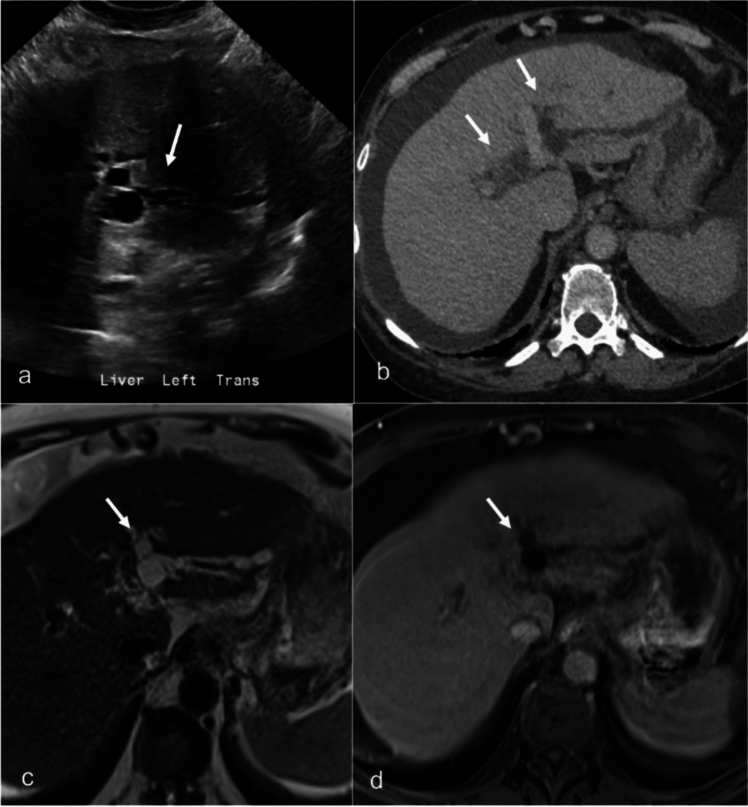
Intraductal papillary neoplasm of the bile duct in a 56-year-old female patient with alcoholic cirrhosis. Grayscale ultrasound images (**a**) shows biliary duct dilation (arrow). Axial CT with contrast (**b**) shows similar finding as US. Axial MRI T2-weighted fat-saturated (**c**) and postcontrast T1 (**d**) images show aneurysmal dilatation of the bile duct (arrows) with distal ductal dilatation. There is no proximal bile duct dilatation (not shown), and no intraduct mass is present. The surgical pathology confirmed the diagnosis

IPN-Bs are classified into four imaging patterns based on their structural characteristics: type 1, an intraductal mass with upstream duct dilatation; type 2, aneurysmal duct dilatation without a visible mass; type 3, both upstream and downstream duct dilatation with an intraductal mass; and type 4, focal cystic dilatation of the bile duct with a papillary mass [39]. Whereas the type 2 pattern is most likely to mimic biliary duct dilation or obstruction, type 4 might overlap with other intrahepatic biliary and peribiliary cystic lesions, particularly MCN. Key features to distinguish from type 4 include direct bile duct communication, intraductal masses and coexistent downstream biliary duct dilation (Fig. 7) [26]. Although IPN-B is considered a premalignant lesion, management is surgical because of the risk of transformation to mucinous adenocarcinoma. This commonly involves partial hepatectomy, bile duct excision, and biliary reconstruction [40]. In nonsurgical candidates, alternatives such as ERCP or peroral cholangioscopic-guided radiofrequency ablation are occasionally considered for palliating biliary obstruction [41].

## Intrahepatic biloma

Intrahepatic bilomas often arise because of biliary necrosis and bile leaks caused by ischemic injury to the bile ducts, commonly seen in liver transplantations or transcatheter interventions via hepatic arteries, such as chemoembolization, radioembolization, and infusion chemotherapy [42]. The necrotic bile duct is seen as dilatation with irregular or beaded margins, leading to development of a focal intrahepatic fluid collection over time [42]. Bilomas can be single or multiple, depending on the extent of biliary necrosis. Typically, these collections are small, sometimes multiple, with well-defined or lobulated margins.

**Fig. 8:**
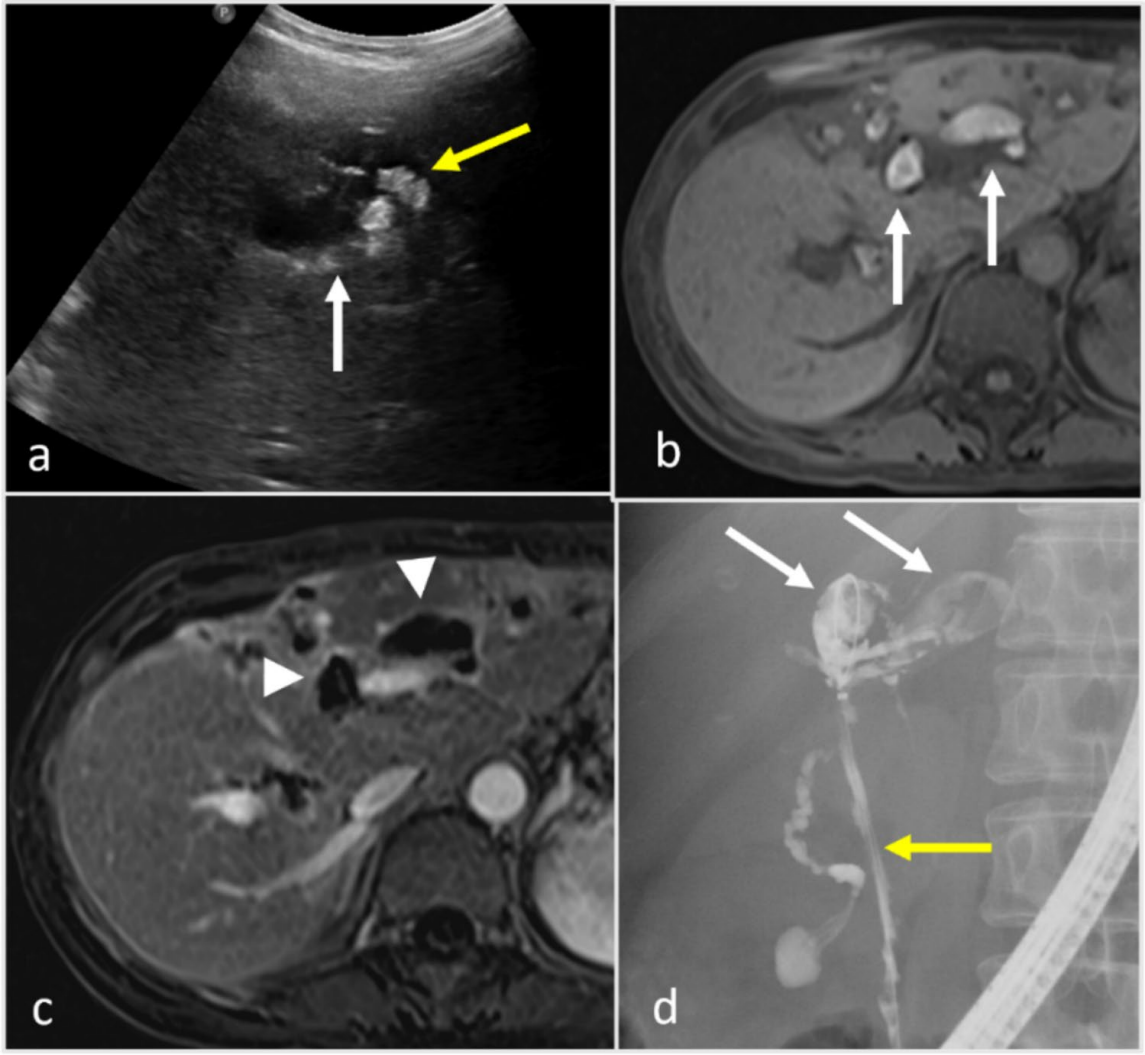
Intrahepatic bilomas in a 60-year-old female with history of Crohn's disease and primary sclerosing cholangitis presenting with elevated bilirubin. Ultrasound of the left hepatic lobe (**a**) shows a dilated intrahepatic duct (white arrow) containing debris (yellow arrow). Subsequent MRI and MRCP (MRCP not shown) showed multiple intrahepatic ducts with dilatation and stricturing. On pre T1-weighted MR imaging (**b**) dilated ducts in the left hepatic lobe are seen containing intrinsic T1 hyperintense debris (white arrows). Subtraction post-contrast MR image (**c**) demonstrates peri-biliary enhancement (white arrowheads) consistent with active cholangitis. The patient underwent ERCP (**d**) which confirmed intrahepatic bilomas with debris (white arrows). Long segment stricturing of the extrahepatic duct can also be visualized (yellow arrow)

On imaging, bilomas appear similar to liver cysts on US and CT. They are hyperintense on T2WI but to a lesser degree than simple cysts on MR (Fig. 8). This distinction arises because the fluid within the damaged bile duct and biloma contains necrotic tissue rather than pure fluid. Delayed imaging with MR hepatobiliary agents such as gadoxetate disodium or a hepatobiliary iminodiacetic acid scan can help confirm a bile leak and communication with the biliary tree. With gadoxetate disodium, biliary excretion and leak can typically be visualized at 20-min post contrast injection in a patient with normal liver function. In cases of slow bile leak flow or hepatic dysfunction, delayed imaging up to one hour may be required for optimal detection [42]. Bilomas may become infected, with imaging features mimicking those of hepatic abscesses.

Management of bilomas varies based on their size and the presence of symptoms. Observation is often sufficient for small, asymptomatic bilomas. However, image-guided percutaneous drainage is recommended for larger collections, with the addition of endoscopic stent drainage if bile duct dilatation is present to expedite bile duct healing and reduce mass effect. Surgical management is considered for cases where percutaneous drainage fails and bile leaks persist [43].

## Abscess

The pathogenesis of hepatic abscesses involves bacterial, fungal, or parasitic infections spreading to the liver hematogenously from distant sites of infection or by direct contamination from an adjacent infection in the abdominal cavity, ascending cholangitis, or penetrating injury. Abscesses occurring in the setting of ascending cholangitis often have a peribiliary distribution and have overlapping imaging features with other forms of biliary or peribiliary cystic disease. In contrast, microabscesses often result from conditions such as septicemia or disseminated fungal infections in which multiple tiny lesions form, particularly in immunocompromised patients [44].

**Fig. 9:**
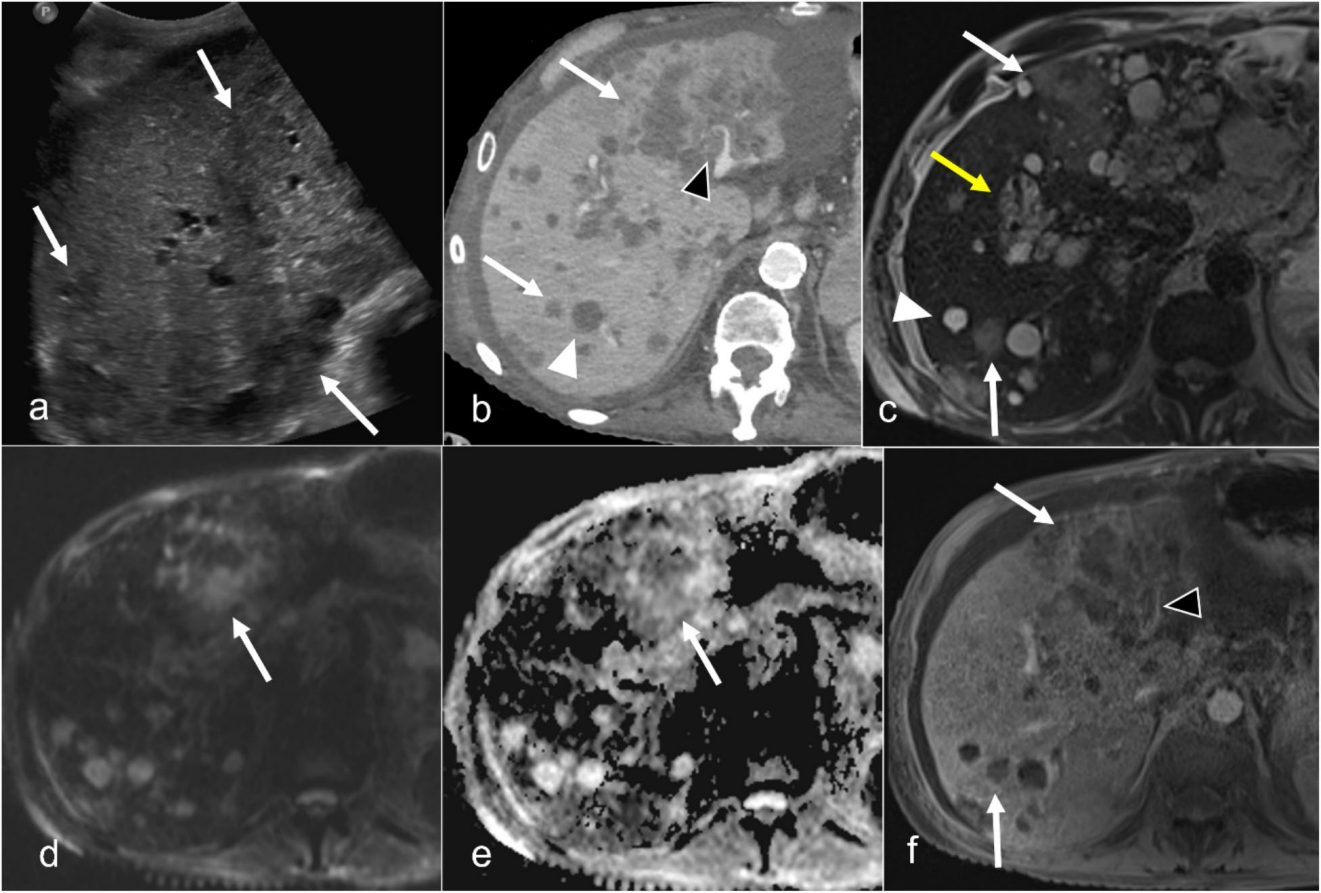
Intrahepatic abscess in a 72-year-old male patient with a history of adult polycystic kidney disease (ADPKD) and end-stage renal disease undergoing radiation treatment for esophageal cancer presents with gram-negative sepsis. Grayscale ultrasound image (**a**) demonstrates multiple areas of low echogenicity (arrows). Axial CT with contrast (**b**) shows mix of simple-appearing cysts (white arrowhead) and low-density lesions with subtle peripheral rim enhancement (arrows). Thrombus is present in the left portal vein (black arrowhead). MRI T2-weighted fat-saturated image (**c**) shows multiple simple cysts (arrowhead) secondary to ADPKD and T2-weighted hypointense lesions (arrows). Additionally, peribiliary cysts are seen (yellow arrow). Diffusion-weighted imaging (**d**) and ADC (**e**) images demonstrate signal restriction on the ADC map (arrow). Postcontrast T1-weighted image (**f**) shows lesions with peripheral rim enhancement and poor enhancement centrally. The left lobe lesion appears ill-defined because of the coalescence of multiple small lesions

On US, hepatic abscesses appear hypoechoic or anechoic lesions with irregular borders. Internal echoes caused by debris or septations may be observed, and increased echogenicity in surrounding tissues can indicate inflammation. Color Doppler imaging typically shows no central flow but may reveal peripheral blood flow because of hyperemia. Detecting microabscesses is challenging because of their small size; they present as multiple hypoechoic areas or hyperechoic due to their small size and opposed walls. CT is particularly effective for diagnosing hepatic abscesses, revealing low-attenuation areas with rim enhancement after contrast administration, a hallmark of abscess formation. The presence of gas within the lesion strongly indicates infection. CT also offers excellent spatial resolution for identifying small microabscesses. On MRI, abscesses typically appear hypointense on T1WI and hyperintense on T2WI, showing diffusion restriction with rim enhancement after contrast administration (Fig. 9). The high-contrast resolution of MRI is especially useful in detecting multiple microabscesses, which are more conspicuous against the surrounding liver parenchyma. The “cluster sign” is seen when multiple small abscesses merge into a single larger abscess cavity, demonstrating peripheral rim enhancement in postcontrast images [9, 44].

Complications of hepatic abscesses include rupture into adjacent structures, resulting in empyema, peritonitis, and sepsis. Treatment typically involves antibiotics and percutaneous drainage of the abscess. Small abscesses (< 5 cm) are generally treated with antibiotics, and percutaneous aspiration is sometimes performed for diagnostic laboratory evaluation. For faster clinical improvement, larger abscesses are managed with percutaneous catheter drainage [44].

## Mimics of biliary and peribiliary cystic lesions

### Hepatic lymphangioma

Hepatic lymphangiomas are rare, benign congenital malformations characterized by dilated lymphatic ducts. These ducts appear as cystic spaces, lined by flattened endothelial cells, and are filled with simple fluid (lymph) [45].

On imaging, hepatic lymphangiomas are often identified as multilocular cystic lesions, although unilocular cysts with internal septations can also be observed. On US, they typically appear as anechoic, avascular, multilocular cystic lesions, often discovered incidentally. Similarly, on contrast-enhanced CT, they present as cystic lesions of low attenuation, with or without thin enhancing septations or fat density in cases where abundant chylous fluid is present. On MRI, they are hypointense on T1WI and hyperintense on T2WI, with hyperintensity similar to that of cerebrospinal fluid. Following contrast administration, septations may show linear enhancement if present. In cases with hemorrhage or proteinaceous fluid, hyperintensity on T1WI and hypointensity on T2WI can be observed [46]. MR lymphography can aid in diagnosis by demonstrating dilated lymphatic ducts [47]. Complications, though rare, include hemorrhage, particularly when the lymphangiomas are large. Management typically involves observation, but surgical intervention may be required in cases of hemorrhage with rupture or diffuse hepatic lymphangiomatosis [48, 49].

### Langerhans cell histiocytosis

Langerhans cell histiocytosis (LCH) is a rare, complex hematologic disorder characterized by the clonal proliferation and accumulation of pathologic bone marrow-derived Langerhans-like cells, a specific type of histiocyte [50]. LCH typically affects young children between 1 and 3 years old and can also affect older children and adults [51]. The disease can be localized to a single site or organ or involve multiple organ systems, commonly the bone, skin, and central nervous system [52]. Hepatobiliary involvement frequently arises in cases of multiorgan disease and is characterized by hepatomegaly and elevated liver enzymes [53].

Hepatobiliary LCH has a particular affinity for the bile ducts and central liver [54]. In its early stages, the disease presents with diffuse liver enlargement, periportal edema, and peribiliary signal abnormalities, typically bandlike or nodular areas that appear hypoechogenic on US, hypodense on CT and show increased signal on T2WI and diffusion-weighted MRI [55]. As the disease progresses, it can lead to the formation of discrete solid nodules and peribiliary destruction, which are hyperechoic on US and hypoenhancing on CT or MRI [56]. Over time, active histiocytic infiltration is replaced by peribiliary fibrosis, resulting in multifocal biliary stricturing and peripheral biliary duct dilation, best visualized with MRI and MRCP [52, 57].

**Fig. 10:**
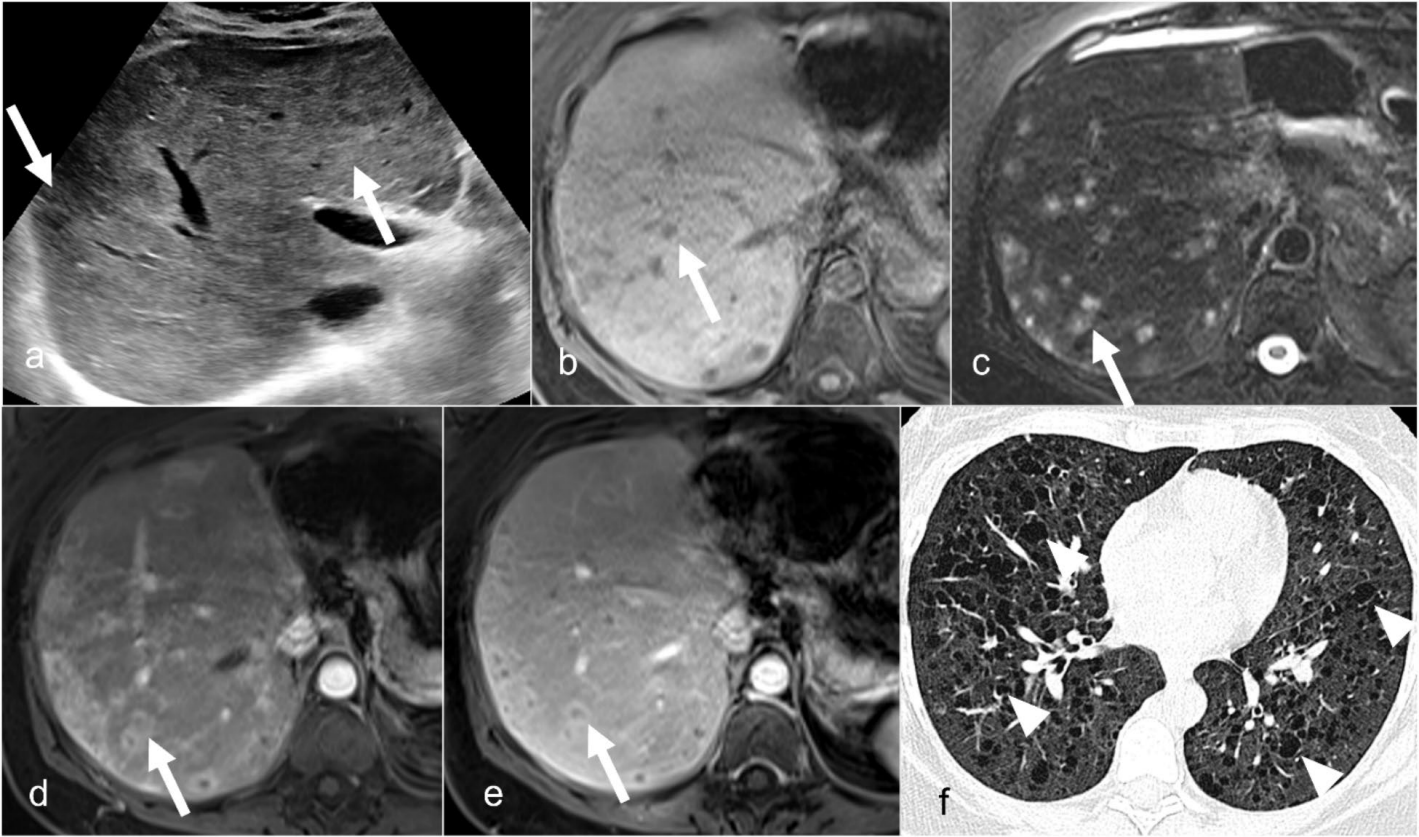
Langerhans cell histiocytosis in a 48-year-old female patient with known involvement of the temporal bone and lung. Grayscale ultrasound image (**a**) demonstrates heterogeneous liver parenchyma with multiple hypoechoic lesions (arrows). Axial MRI T1-weighted (**b**), T2-weighted fat-saturated (**c**), and postcontrast T1-weighted arterial and venous phase (**d**, **e**) images show T1 hypointense and T2-weighted hyperintense subcentimeter lesion with peripheral rim enhancement (arrows). Axial CT of the lung shows multiple small thin-walled cysts (arrowheads)

LCH, especially in its early stages, can mimic peribiliary or biliary cystic disease on CT. However, it is distinguishable from these conditions on US or MRI–MRCP (Fig. 10). Treatment for multiorgan or hepatobiliary LCH involves a combination of steroids and chemotherapy [58]. In severe cases in which sclerosing cholangitis leads to biliary cirrhosis, liver transplantation may become necessary [59].

### Neurofibromatosis

Neurofibromatosis type 1 (NF1), also known as von Recklinghausen disease, is a genetic disorder linked to NF1 tumor suppressor gene mutations [60]. Multiple physical manifestations, including café-au-lait spots, axillary and inguinal freckling, optic pathway gliomas, and neurofibromas, characterize this autosomal dominant condition. Plexiform neurofibroma is a variant of neurofibroma characterized by growth along the length of the nerves, affecting nerve fascicles and branches [61]. Although commonly found in the abdomen and pelvis, their occurrence in the porta hepatis is rare [62].

**Fig. 11:**
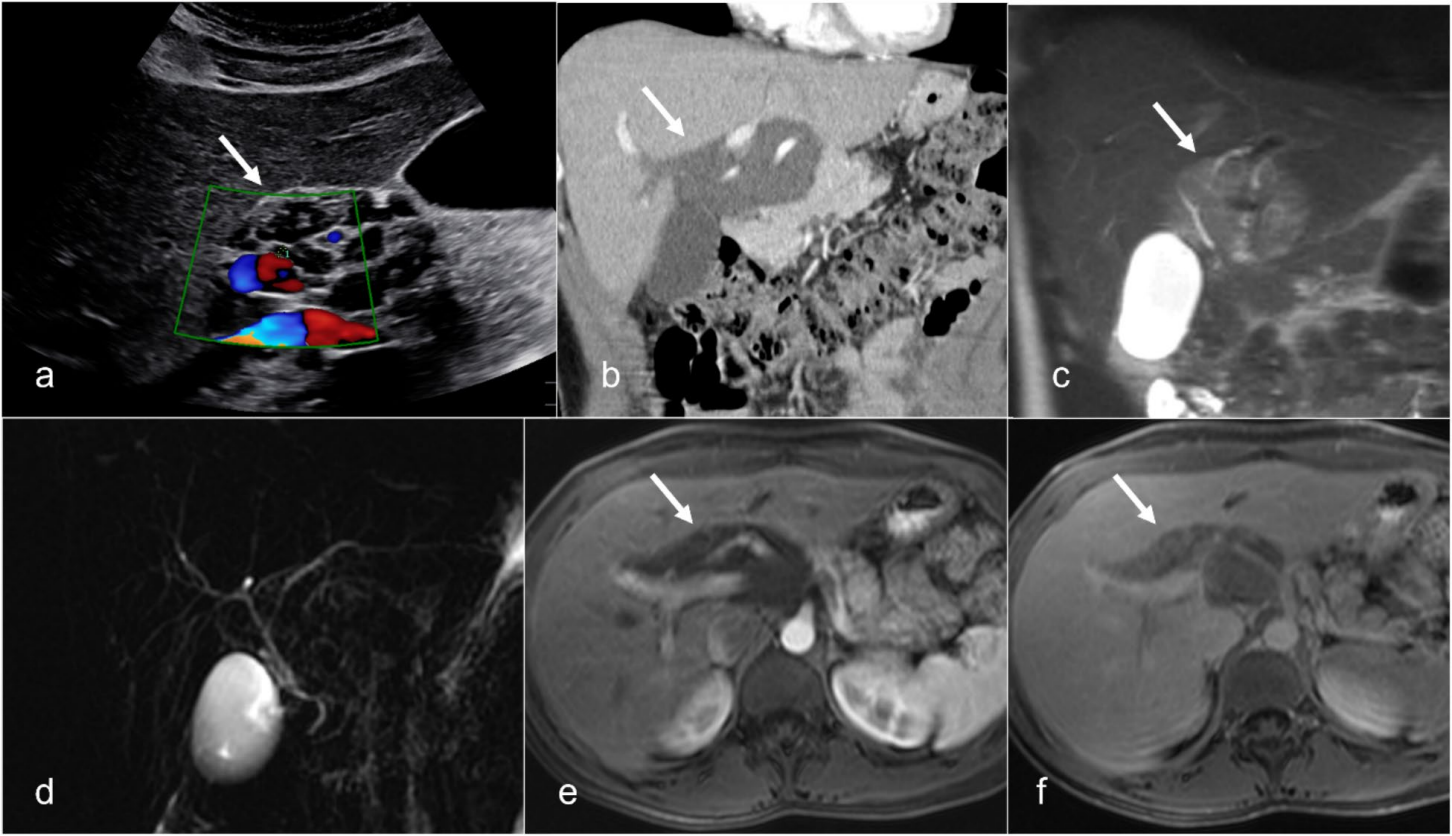
Neurofibromatosis in a 23-year-old patient with abdominal pain. Color Doppler US image (**a**) shows anechoic appearing cystic lesion with no blood flow. Coronal CT with contrast (**b**) demonstrates a poorly enhancing infiltrating lesion along the hepatic vasculature. Coronal MRI T2-weighted fat-saturated (**c**) images show T2-weighted hyperintense lesion (arrow) infiltrating along the bile ducts. MR cholangiopancreatography maximum intensity projection image (**d**) shows no bile duct dilatation. Postcontrast T1-weighted arterial (**e**) and delayed phase (**f**) shows delayed enhancement of the lesion (arrow). The lesion was biopsied to confirm the finding of a plexiform neurofibroma

Because of the myxoid component, plexiform neurofibromas have low density on CT, hyperintensity on T2W MRI, and are relatively hypoenhancing [63]. These tumors often present as multinodular and confluent masses that can exert mass effect on adjacent structures. A distinctive feature is the “target sign” on MRI, in which the periphery of a nodule is hyperintense because of the myxoid stroma, and the center has a low T2 signal because of the fibrotic component [64]. When located in the hepatic hilum, these neurofibromas can resemble peribiliary cystic disease on CT because of their low attenuation but can easily be identified as solid on contrast-enhanced MRI (Fig. 11). Although benign, plexiform neurofibromas carry a significant risk of transforming into malignant peripheral nerve sheath tumors [65]. Management strategies range from surveillance of smaller lesions to using treatment with selumetinib or surgery for larger lesions [66].

### Vascular entities

**Fig. 12:**
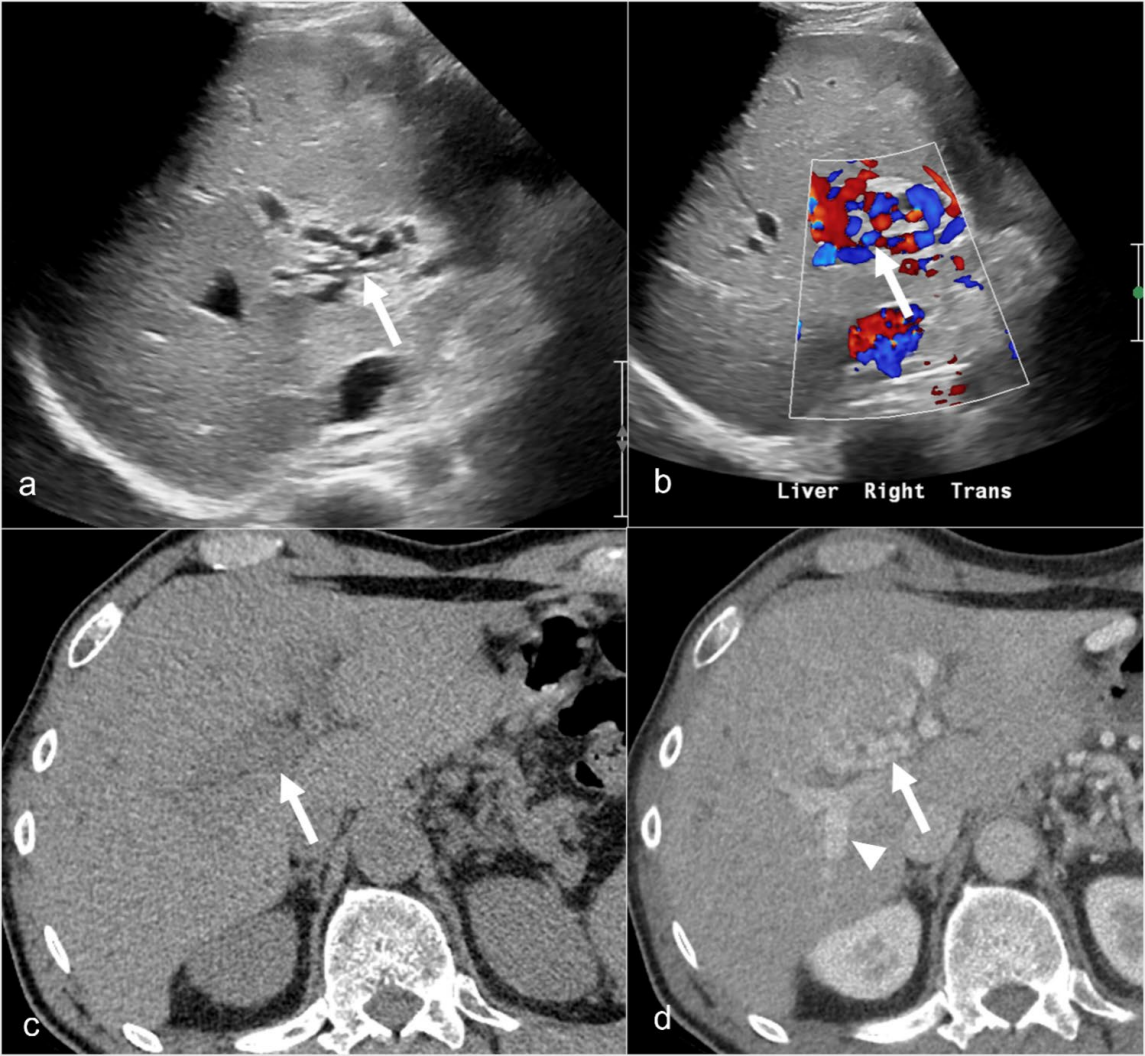
Cavernous transformation of the portal vein in a 34-year-old male patient with recurrent abdominal pain. Grayscale (**a**) and color Doppler (**b**) Ultrasound image demonstrate anechoic tortuous appearing cystic lesion (arrow). Blood flow is present on color Doppler (arrow). Axial non-contrast CT (**c**) demonstrates low density area in the region of portal vein (arrow). Axial contrast-enhanced CT (**d**) demonstrates tortuous veins secondary to cavernous transformation of the portal vein in the setting of necrotizing pancreatitis.

Vascular entities such as cavernous transformation of the portal vein, aneurysm, and pseudoaneurysm can mimic peribiliary cystic lesions on noncontrast cross-sectional imaging and grayscale US, so they may present diagnostic challenges. Cavernous transformation, characterized by the formation of a network of tortuous veins at the portal vein’s location, can mimic peribiliary cysts because of their cystic appearance on non-contrast imaging. This condition typically arises as a sequelae of portal vein thrombosis. The radiologic similarity to peribiliary cysts is marked by the presence of multiple rounded structures, which are actually varices, around the bile ducts. Color Doppler US and enhancement pattern in contrast-enhanced CT and MRI help identify the vascular nature of these multiple tortuous small varices juxtaposed to bile ducts (Fig. 12) [67]. Rarely, portal biliopathy can occur where extrinsic compression on the bile ducts is caused by peribiliary collateral, resulting in biliary dilation and stricturing [57]. Patients with resultant biliary obstruction or cholangitis may require biliary drainage or even portal decompression with a portosystemic shunt [68].

Intrahepatic aneurysms and pseudoaneurysms in the hepatic arteries can also be mistaken for peribiliary cysts. An aneurysm involves a localized dilation of an artery, which may appear as a round, fluid-filled structure adjacent to bile ducts, thereby simulating the appearance of peribiliary cysts. They are often found incidentally but may be associated with fibromuscular dysplasia, vasculitis, systemic lupus erythematosus or polyarteritis nodosa, Takayasu arteritis, and Wegener granulomatosis [69].

A pseudoaneurysm, which results from arterial wall injury with subsequent blood collection that is contained by surrounding tissues, also presents a cyst like appearance on US. Similar to cavernous transformation, the vascular nature of these findings is well depicted on color Doppler US and contrast-enhanced CT and MRI. CT angiography is the imaging modality of choice for diagnosing aneurysms and pseudoaneurysms. Gastrointestinal (GI) bleeding and hemobilia may be present when the pseudoaneurysm is in communication with the bile ducts or GI tract [69]. Large aneurysms (i.e., > 2 cm) and pseudoaneurysms are almost always managed with an arterial stent or coiling at the aneurysm or pseudoaneurysm neck [70].

## Challenges in the diagnosis of biliary and peribiliary cystic lesions

Identifying whether a cystic lesion arises from the hepatic artery, portal vein, bile ducts, or lymphatics can be, at times, difficult by a single imaging modality or imaging performed without intravenous contrast. Therefore, a multimodality approach or MRI with MRCP usually helps narrow the differential diagnosis. An important value of imaging is to confidently distinguish benign from malignant or premalignant lesions.

**Fig. 13:**
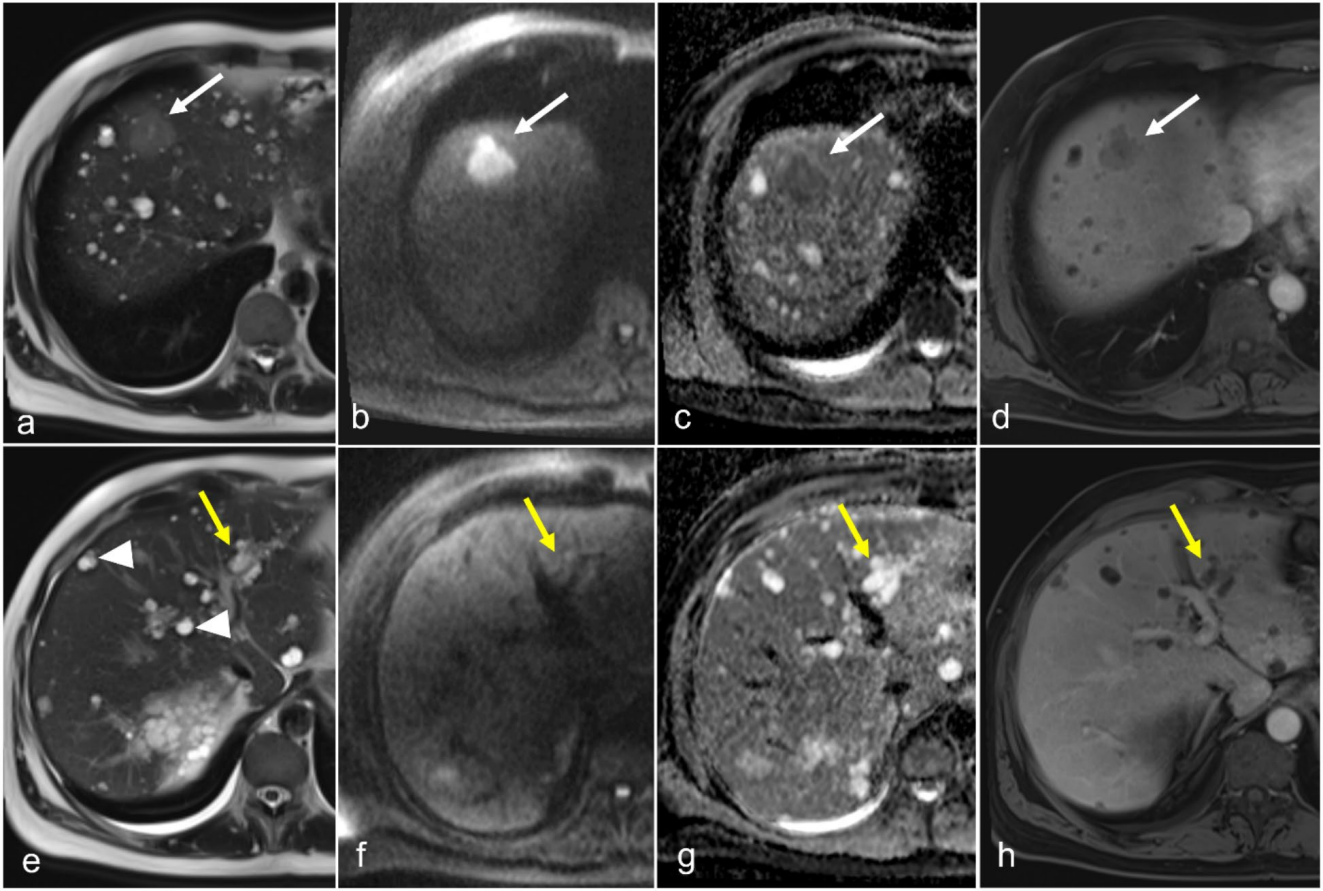
Cystic metastasis in a patient with a history of autosomal dominant polycystic kidney disease and sigmoid carcinoma with solitary liver metastases. Axial T2-weighted images (**a**) demonstrate a mild T2-weighted hyperintense mass (arrow) with focus of central necrosis in hepatic segment 8 near the dome. The mass demonstrates increased signal intensity on diffusion-weighted images (**b**) and appears dark on the ADC maps (**c**) representing restricted diffusion. On late arterial phase postcontrast T1-weighted imaging (**d**), the mass demonstrates mild central enhancement with thickened and irregular peripheral enhancement (arrow). The mass (white arrows) demonstrates a more distinctive feature from liver parenchymal cysts (arrowheads) and the peribiliary cysts in the left hepatic lobe (yellow arrows). The cysts demonstrate more intense T2-weighted hyperintensity (**e**); no restricted diffusion (**f**, **g**); and thin, smooth peripheral enhancement (**h**). The segment 8 mass is a biopsy-proven adenocarcinoma metastasis

Cystic metastases can arise in the peribiliary space because of seeding along the portal tract and can mimic peribiliary cystic lesions. Hence, tumors that arise from the GI tract (GI stromal tumors) or the pancreas (neuroendocrine tumors), colorectal metastases (Fig. 13), and melanoma can appear cystic in the peribiliary space because of degeneration, outgrowing their vascular supply, or becoming necrotic. Often, the walls appear irregular and thickened. Other features that suggest malignancy include mural nodularity, especially if the mural nodularity enhances; presence of multiple locules; and association with biliary ductal dilatation.

**Fig. 14:**
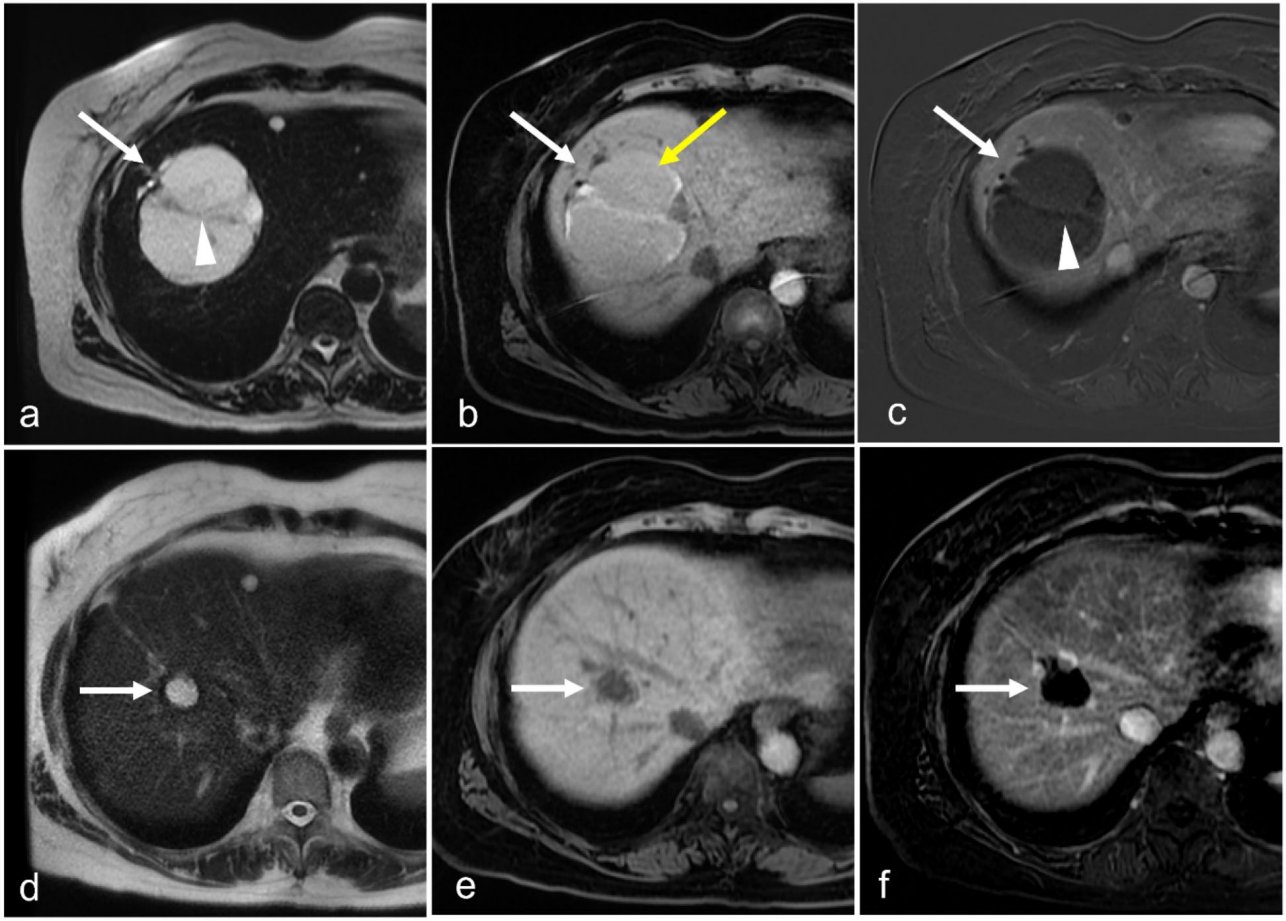
Hemorrhagic cyst in a 68-year-old female patient with history of breast cancer and elevated alkaline phosphatase level. Axial T2-weighted MR image (**a**) demonstrates a large right hepatic lobe cyst with a thickened-appearing septation (white arrowhead) and upstream biliary ductal dilatation (white arrow). Precontrast axial T1-weighted image (**b**) demonstrates a T1-hyperintense rim and septa suggesting methemoglobin. Mild T1 hyperintense fluid within the cyst suggests proteinaceous or hemorrhagic contents (yellow arrow). Subtraction postcontrast axial T1-weighted image in the late arterial phase (**c**) demonstrates minimal enhancement of the wall (arrow) and septa (arrowhead). The patient underwent biopsy, which was nondiagnostic. Because of the presence of biliary ductal dilatation and concern for biliary cystadenoma, surgery was recommended. The patient chose to defer the surgery and observe. Serial imaging every 3 to 6 months demonstrated a decrease in the size of the cyst. The patient’s alkaline phosphatase levels continued to improve. Axial T2-weighted imaging performed 16 months after the initial scan (**d**) demonstrates a substantial decrease in the cyst size and nearly resolved duct dilatation (white arrow). Precontrast axial T1-weighted image (**e**) demonstrates resolution of the methemoglobin and proteinaceous or hemorrhagic contents. Subtraction postcontrast axial image in the late arterial phase (**f**) demonstrates no enhancing septa or nodularity. Overall, the findings were believed to represent resolving hemorrhage within a peribiliary cyst

Hemorrhage within a cystic lesion can be a confounder in differentiating underlying malignancy from benign disease. Hemorrhage can create fibrinlike deposition mimicking septa (Fig. 14). Although associated biliary ductal dilatation may suggest malignancy, this feature has limited specificity as intrahepatic ducts can often dilate because of mass effect (Fig. 14a). Sampling errors and nondiagnostic biopsies can create dilemmas in management. Often, the best available tool is serial imaging to evaluate the size and any morphologic changes to suggest a malignant transformation of indeterminate cysts (Fig. 14d–f).

## Conclusion

Intrahepatic biliary and peribiliary cystic lesions range from benign congenital cysts to complex lesions with malignant potential. Often asymptomatic and incidentally discovered, accurate characterization through imaging is essential. Imaging enhances diagnostic confidence by distinguishing benign from malignant lesions and detecting complications such as cholangitis, and biliary obstruction, which are helpful for guiding clinical management. A comprehensive understanding of these cystic lesions allows for individualized treatment strategies, from conservative monitoring to more aggressive interventions. Effective management relies on imaging evaluation and interdisciplinary collaboration, ensuring optimal patient outcomes while minimizing unnecessary procedures.

## Data Availability

No datasets were generated or analysed during the current study.
